# Modeling Atrial Fibrillation in a Human Heart Macrophage Assembloid

**DOI:** 10.51894/001c.143897

**Published:** 2025-09-30

**Authors:** Colin O’Hem, Sammantha Caywood, Weiheng Cao, Mia Dionise, Hussain Basrai, Milana Skoric, George Boulos, Nagib Chalfoun, Aitor Aguirre

**Affiliations:** 1 College of Osteopathic Medicine Michigan State University

## 13

### INTRODUCTION

Cardiac arrhythmias are abnormal heart rhythms that can lead to palpitations, stroke, heart failure, and sudden cardiac arrest. Atrial fibrillation (AF) is the most prevalent cardiac arrhythmia, characterized by irregular atrial firing. Recent studies have linked inflammation—particularly in cases of myocarditis, pericarditis, and sepsis—to new-onset AF. One known mechanism of inflammation-induced AF, identified in animals, is the activation of the NLRP3 inflammasome, a protein complex that forms inside atrial cardiomyocytes. While NLRP3 inflammasome activation has been observed in post-mortem human hearts with chronic and paroxysmal AF, its causal role in human AF remains unproven.

### OBJECTIVES

We hypothesized that activation of the NLRP3 inflammasome induces AF in human heart macrophage assembloids (hHMAs), an in vitro pluripotent stem cell derived human heart organoid with integrated tissue resident macrophages.

### METHODS

To model inflammation-induced AF, we administered known pro-inflammatory activators of the NLRP3 inflammasome, including LPS, IFN-γ, and IL-1β, at physiological blood concentrations to hHMAs. We utilized RT-qPCR to assess gene expression, confocal microscopy to localize NLRP3-inflammasome protein components, FluoVolt live-cell imaging to visualize conductance patterns in atrial cardiomyocytes, and phase-contrast microscopy to assess assembloid morphology and contractility.

### RESULTS

NLRP3 inflammasome activation induced spontaneous irregular rhythms in a significant proportion of hHMAs, leading to irregular conductance patterns in atrial cardiomyocytes. These findings confirm that the NLRP3 inflammasome is activated in atrial cardiomyocytes of arrhythmic hHMAs.

### CONCLUSIONS

This inflammation-AF hHMA model provides a novel platform to study the mechanistic link between inflammation and AF. By closely mimicking human AF, this system has the potential to advance pharmacological development for AF and enable efficient preclinical trials for anti-arrhythmic therapies.

